# Rheometric Non-Isothermal Gelatinization Kinetics of Chickpea Flour-Based Gluten-Free Muffin Batters with Added Biopolymers

**DOI:** 10.3390/foods6010003

**Published:** 2017-01-02

**Authors:** María Dolores Alvarez, Francisco Javier Cuesta, Beatriz Herranz, Wenceslao Canet

**Affiliations:** 1 Department of Characterization, Quality, and Safety, Institute of Food Science, Technology, and Nutrition (ICTAN-CSIC), José Antonio Novais 10, 28040 Madrid, Spain; beatriz.herranz@ictan.csic.es (B.H.); wenceslao@ictan.csic.es (W.C.); 2 Department of Products, Institute of Food Science, Technology and Nutrition (ICTAN-CSIC), José Antonio Novais 10, 28040 Madrid, Spain; fcb@ictan.csic.es

**Keywords:** gluten-free, chickpea flour, non-isothermal heating, elastic modulus, reaction kinetics, activation energy, gelatinization, food process modeling

## Abstract

An attempt was made to analyze the elastic modulus (*G*′) of chickpea flour (CF)-based muffin batters made with CF alone and with added biopolymers (whey protein (WP), xanthan gum (XG), inulin (INL), and their blends) in order to evaluate their suitability to be a wheat flour (WF) substitute in muffins, and to model the heat-induced gelatinization of batters under non-isothermal heating condition from 25 °C to 90 °C. A rheological approach is proposed to determine the kinetic parameters (reaction order (*n*), frequency factor (*k*_0_), and activation energy (*E*_a_)) using linearly-increasing temperature. Zero-order reaction kinetics adequately described batter gelatinization process, therefore assuming a constant rate independent of the initial *G*′ value. The change of the derivative of *G*′ with respect to time (*dG*′/*dt*) versus temperature is described by one exponential function with activation energies ranging from 118 to 180 kJ·mol^−1^. Control wheat gluten batter, with higher and lower starch and protein contents, respectively, than CF-based batters, exhibited the highest *E*_a_ value. Formulation of CF-based gluten-free batters with starch and protein contents closer to the levels of WF-based batter could be a strategy to decrease differences in kinetic parameters of muffin batters and, therefore, in technological characteristics of baked muffins.

## 1. Introduction

Muffins are a popular breakfast or afternoon snack food, which are highly appreciated by consumers due to their good taste and soft texture [1]. Traditionally, a muffin recipe is mainly composed of wheat flour (WF), sugar, vegetal oil, egg, and milk [2]. For this reason, persons with celiac disease (CD) are unable to consume this type of baked product since they are made with WF [1]. In addition, today, there is an increasing number of people interested in wheat-free foods motivated by the desire to avoid wheat in the diet [3]. Most of gluten-free muffin, cake, or cupcake recipes contain rice flour as the principal ingredient [1,3,4,5,6] or other starch sources, such as corn, potato, or wheat [3,7]. However, many gluten-free products available on the market are often of poor technological quality, exhibiting low volume, poor color, and crumbling crumb, besides great variation in the nutrient composition, with low protein and high fat contents [8], particularly when compared to their wheat counterparts [9]. On the other hand, baked muffins are characterized by a typical alveolar-porous structure and high volume, which give a spongy texture. In turn, a muffin batter may well be defined as a “cellular system” [10], in which the continuous semisolid matrix formed by a complex fat-in-water emulsion could be considered a multiphase system containing ungelatinized starch granules, oil droplets, proteins, sucrose, etc. 

The United Nations declared 2016 the International Year of Pulses (IYP 2016). The hope of IYP 2016 is to position pulses as primary sources of protein and other essential nutrients, leading to dietary uptake. Chickpea (*Cicer arietinum* L.) is a legume rich in protein, dietary fiber, carbohydrates, folate and trace minerals (Fe, Mo, Mn) [11]. The functional properties of chickpea protein provided good baking characteristics in gluten-free and wheat breads elaborated with chickpea flour (CF) [12]. Therefore, the use of CF would be a big challenge as alternative to WF in the production of high-quality gluten-free baked muffins. However, to obtain good technological quality, a stable semisolid batter lodging many tiny air bubbles is required [13,14]. 

Incorporation of hydrocolloids such as gums and proteins from gluten-free sources has been suggested to improve gluten-free muffin quality characteristics [15]. For example, a combination of erythritol with xanthan gum (XG) and double quantities of leavening agent was effective in improving the texture properties of the muffins, reducing their hardness, which this was associated with a significant increase in batter air content and in muffin height, volume, and number of bubbles in comparison with the employment of erythritol alone [14]. 

Modeling aspects of food processing can contribute for the improvement of the food industry. However, there is a necessity of mathematical modeling in the food processing operations to achieve sustainable processing industry. Baking is a decisive stage in the production of bakery products, in general—muffins, in particular—for most of the quality attributes of the final products depend on it [16]. The authors just cited established the kinetics of muffin crust color development during baking in order to evaluate the feasibility of this kinetic model to predict the baking times.

To investigate the structural changes taking place in different muffin batters during heating in the oven, the linear viscoelastic properties can be studied during non-isothermal heating (temperature sweep), trying to simulate the batter’s behavior in the oven [13,14,17,18]. Both rheology and differential scanning calorimetry (DSC) provide information at physical and macroscopic levels indicating changes associated with gelatinization or denaturation processes [19]. However, sometimes DSC measurement is not able to detect gelatinization temperature by providing required endothermic curve [20]. On the other hand, rheometric measurement (small amplitude oscillatory shear (SAOS) measurement) has been found to be more precise to detect gelatinization temperature during non-isothermal heating than DSC, providing more authentic information on gelatinization and also reaction kinetics of gelatinization process [20,21,22].

The reaction kinetics in food systems have commonly been studied under isothermal heating conditions. However, the isothermal process has some practical limitations, especially when dealing with samples that are difficult to heat instantaneously to testing temperatures [22]. Some studies have been published for different food systems on these kinetic approaches under non-isothermal conditions [20,21,22,23,24,25], which allows parameter estimation from a single experiment where temperature is varied over the range of interest, and samples are taken at various intervals. Therefore, it would be interesting to study the influence of non-isothermal heating on muffin batter rheology. Such studies with SAOS measurement techniques in the linear viscoelastic range would provide a broader insight to the gelatinization kinetics. The order of reaction (*n*) and the energy required to achieve gelatinization (activation energy, *E*_a_) can be calculated from thermorheological data. These studies could provide a good insight into gelatinization and gelling mechanisms of muffin batters, as well as useful data for potential replacement of WF with CF in gluten-free muffin formulation.

The objective of this work was to evaluate and model the non-isothermal gelatinization kinetics of CF-based gluten-free muffin batters with and without added biopolymers by a rheological approach, with a view to providing kinetic parameters that could contribute to effective total replacement of WF in muffin formulation.

## 2. Materials and Methods

### 2.1. Material

Muffin ingredients were commercial WF and CF (11.5% and 9.9% moisture and 10.2% and 19.4% protein, respectively), both donated by García del Valle flour milling company (Soria, Spain), pasteurized liquid whole yolk (Ovopak, Seville, Spain), sucrose (AB Azucarera Iberia S.L., Madrid, Spain), salt (sodium chloride), ultra-high-temperature whole milk (Pascual, Burgos, Spain), refined sunflower oil (Koipesol, Madrid, Spain), citric acid anhydrous, and sodium hydrogen carbonate (Panreac Química S.L.U., Barcelona, Spain). Microparticulated WP concentrate (53% protein, SIMPLESSE 100 (E)) and XG (Keltrol F (E)) were donated by Premium Ingredients, S.L. (Girona, Spain), while inulin (INL) was a “long-chain” INL with trade name Orafti HP (BENEO-Orafti, Tienen, Belgium).

### 2.2. Gluten-Free Muffin Batter Making

One batter formulation was prepared as control with WF alone (Table 1). Then, CF-based muffin batters were made without adding any biopolymer by replacing all of the WF with CF or by replacing part of the CF with WP, XG, INL, and their blends. Samples were formulated and identified as shown in Table 1.

The batter was prepared as described by Martínez-Cervera et al. [13,14] with some modifications, using a KPM5 professional mixer (Kitchen Aid, St. Joseph, MI, USA). When added, WP and INL at 5, 10, and 15 g 100 g^−1^ of CF were previously dissolved in the milk at 75 °C for 15 min, while XG (0.25, 0.50, and 1 g 100 g^−1^ of CF) was incorporated as a dry powder. The batters were all kept at 25 °C for 60 min before measurements. Each batter formulation was prepared at least three times (three batches), on different days.

### 2.3. Rheological Measurements of Muffin Batter

Small-amplitude oscillatory shear (SAOS) tests were carried out with a Bohlin CVR 50 controlled stress rheometer (Bohlin Instruments Ltd., Cirencester, UK), using parallel-plate geometry (40 mm diameter) and gap (1-mm). Samples were allowed to rest for 15 min before analysis to ensure both thermal and mechanical equilibrium, and they were covered with a thin film of Vaseline oil (PRS-Codex) to avoid evaporation. Temperature was controlled to within 0.1 °C by Peltier elements in the lower plate. Temperature sweep tests were performed from 25 °C to 90 °C at a linear heating rate (1.6 °C·min^−1^). Frequency was set at 0.1 Hz, maintaining the shear stress signal at the minimum value provided by the Bohlin rheometer for parallel-plate geometry (2.98 Pa). During non-isothermal heating, values of elastic modulus (*G*′, Pa) and viscous modulus (*G*′′, Pa) were recorded. Three replicates were carried out with batters prepared on three different days (*n* = 9).

### 2.4. Kinetic Modeling of Non-Isothermal Rheological Data

The general form for non-isothermal kinetics combining reaction rate, time-temperature profile, and Arrhenius relationship can be written as:

(1) $$\int_{C_{0}}^{C}\frac{dC}{C^{n}}=k_{0}\int_{0}^{t}\exp\left(-\frac{E_{a}}{RT}\right)dt$$

where *C*_0_ is the concentration at zero time, *C* the concentration at time *t*, *k*_0_ the pre-exponential or frequency factor (Pa^(1−n)^·s^−1^), *E*_a_ the activation energy (kJ·mol^−1^), *T* the absolute temperature (K), and *R* the universal gas constant (8.314 J·mol^−1^·K^−1^).

In fact, according to Rhim et al. [26] for *n*th order decomposition reaction the rate is described by:

(2) $$-\frac{dC}{dt}=kC^{n}$$

where *C* is the concentration at time *t* and *k* is a temperature dependent parameter (Pa^(1−n)^·s^−1^).

Heating rate affects the evaluation of this general form of the equation [21]. The temperature dependency of the reaction rate parameter k is well represented by the Arrhenius relationship:

(3) $$k=k_{0}e^{\left(\frac{-Ea}{RT}\right)}$$

*k*_0_ being the pre-exponential or frequency factor (Pa^(1−n)^·s^−1^), *E*_a_ the activation energy (kJ·mol^−1^), *T* the absolute temperature (K), and *R* the universal gas constant (8.314 J·mol^−1^·K^−1^).

So that the general form for non-isothermal kinetics is (combining Equations (2) and (3)):

(4) $$-\frac{dC}{dt}=k_{0}C^{n}e^{\left(\frac{-E_{a}}{RT}\right)}$$

By integrating, Equation (1) is obtained.

In accordance with previous works [19,20,21,22,23,24], the kinetic parameters *E*_a_ and *k*_0_ can be estimated from Equation (4). Indeed, in semi-logarithmic plot, Equation (4) can be rewritten as:

(5) $$\ln\left(-\frac{1}{C^{n}}\frac{dC}{dt}\right)=\ln k_{0}-\left(\frac{E_{a}}{R}\right)\left(\frac{1}{T}\right)$$

The kinetic parameters *E*_a_ and *k*_0_ are estimated from the linear regression of Equation (5).

The non-isothermal kinetic relation based on the experimental data and regression analysis was carried out following the steps described by Rhim et al. [26] for a linearly increasing temperature system.

### 2.5. Statistical Analysis of Kinetic Parameters

A one-way analysis of variance (ANOVA) was used to evaluate the effect of the formulation on the kinetic parameters during non-isothermal heating of muffin batters. Minimum significant differences were calculated by Fisher’s least significant difference (LSD) tests at significance level 0.05. Pearson product-moment correlations were determined with significance levels based on the Student’s distribution. Statistical analyses were carried out using the SPSS 19.0 statistical software package (SPSS Inc., Chicago, IL, USA).

## 3. Results and Discussion

### 3.1. Effect of Non-Isothermal Heating on Viscoelastic Rheological Properties

**Figure 1:**
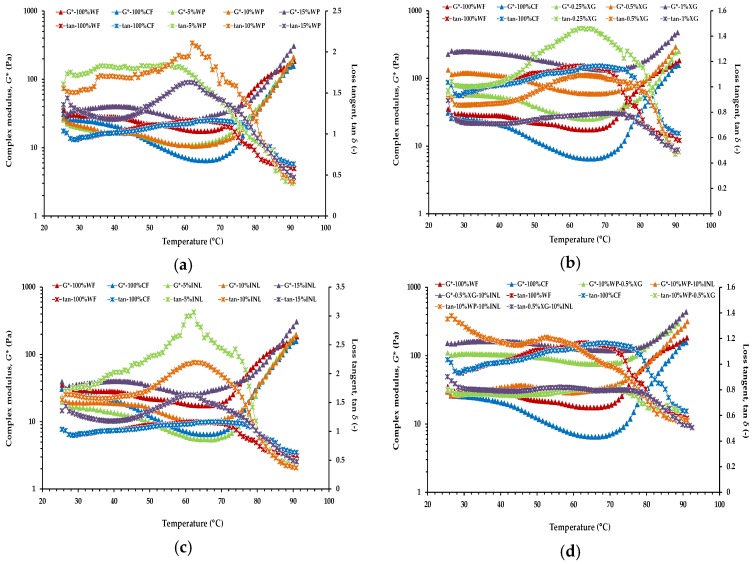
Complex modulus (G*, Pa) and loss tangent (tan *δ*, -) during non-isothermal heating for the control wheat flour (WF)-based batter and chickpea flour (CF)-based batters prepared with CF alone and with partial replacement of CF with whey protein (WP) alone at 5%, 10%, and 15% levels (**a**), with xanthan gum (XG) alone at 0.25%, 0.5%, and 1% levels (**b**), with inulin (INL) alone at 5%, 10%, and 15% levels (**c**), and with WP at 10% level blended with XG at the 0.5% level, WP at the 10% level, blended with INL at the 10% level, and XG at the 0.5% level, and blended with INL at the 10% level (**d**). Mean values of nine measurements.

The structural changes that occur during baking are considered determining factors in bubble formation and stability and, therefore, determine the final microstructure and texture of the baked product [27]. In this regard, Figure 1 shows the temperature sweep test results in terms of complex modulus (G*) and loss tangent (tan *δ*) values of the CF-based muffin formulations with added biopolymers and their blends together 100% WF and 100% CF batters during non-isothermal heating from 25 °C to 90 °C. G* is a combination of the elastic modulus *G*′ and the dissipative one *G*′′ [28]. During heating, practically all of the samples showed an initial decrease of the batter moduli, when heated up to the gelatinization temperatures. Heating patterns of batters made with WP and INL alone (at 5%, 10%, and 15%) and 100% WF and 100% CF batters were quite similar (Figure 1a,c). It can be seen that at the beginning of heating of many of these muffin batters, values of tan *δ* above unity indicates the prevalence of liquid-like behavior, the elastic modulus being lower than the dissipative one. It is noteworthy that in batters made with WP and INL alone, tan *δ* increases its value showing a more pronounced decrease of *G*′ with respect to *G*′′ in the early stage of heating. On the contrary, the CF-based batters with partial replacement of CF with XG alone at 1% level (Figure 1b), as well as those with partial replacement of CF with XG at 0.5% level blended with INL or WP at 10% level (Figure 1d), had values of tan *δ* below one during all the temperature range studied, reflecting their more solid-like behavior.

Therefore, the initial increase in temperature produced a decrease in the values of the dynamic moduli, associated with a decrease in viscoelasticity (higher tan *δ* value) until they reached an inflexion point. As observed by Migliori et al. [28] and Chen et al. [29], this initial moduli decrease up to the onset of gelatinization (batter softening) may be attributed to a kinetic effect caused by the temperature increase that tends to weaken the structure. As long as the kinetic effect prevails the strength of the interactions decreases and a weakening of the structure is observed. In addition, in this study, all of the muffin batters contained whole egg at the same level. However, it was showed that no change in interacting rheological units is determined by egg level when the temperature is bellow 50 °C [28].

In turn, Matos et al. [1] reported that the decrease in modulus values up to the temperature of 45 °C is related to the release of CO_2_ formed in the batter, which is diffused into occluded air cells and expanded and, hence, reduces batter density. On the other hand, note that the decrease in G* was much more evident in 100% CF than in 100% WF after 45 °C. When no structure is formed in the batter, the prevailing effect below 50 °C is batter thickening caused by starch [28]. The lower carbohydrate content of CF might also influence the more significant G* decrease observed in 100% CF batter [30].

As the temperature is increased above inflexion points (gel point, (*T*_gel_)), G* sharply increases up to 90 °C and tan *δ* drops below one (Figure 1). The gel point, most commonly defined as the temperature at which *G*′ and *G*′′ intersect [31], generally occurs as a result of the early stage of starch gelatinization [22]; this stage corresponds to when starch granules swell and melt. It can be observed that in the batter made with CF alone (100% CF), there is a noticeable delay in the temperature of cross-over of both moduli when compared with the control batter (100% WF). The delay in starch gelatinization in 100% CF could be crucial to avoid earlier thermosetting and give enough time for appropriate air and vapor expansion during baking [13,14]. Using DSC, both starch and protein peaks could be distinguished clearly, when a sample size of 4.5 mg of CF (~10% moisture) in 23 mg of water was used [32]. Temperatures of starch peak gelatinization and protein denaturation were found to be in the ranges 75–90 °C and 100–120 °C, respectively. It can be expected, because of the temperature difference in starch and protein conformation disruption, that the strengthening of the network in the muffin batters will, firstly, be based on starch gelatinization and that protein denaturation will have an input later and at higher temperatures and times. Therefore, in this study, it is assumed that, mainly, the process of starch gelatinization governs the evolution of the elastic modulus in the muffin batters during heating between 25 °C and 90 °C. Meares et al. [32] reported that, within CF, there is also a small amount of lipids in the flour, which can interact with amylose in starch to give a reversible endothermic peak on heating. 

On the other hand, the incorporation of XG (individually or combined with INL or WP) into the 100% CF batter produced a significant increase in the viscoelastic properties during the whole heating period (Figure 1b,d). For batters with partial replacement of CF with XG, alone (at 1%) or blended with either WP or INL, *G*′ was found to be consistently higher than *G*′′ in the studied temperature range, which indicates a more elastic character during heating of CF-based batters enriched with XG. A previous study also showed that batters with added XG, alone (0.5% XG and 1% XG) or combined with WP or INL (10% WP–0.5% XG and 0.5% XG-10% INL), had a significant increase in conformational stability (higher critical shear stress (*σ*_max_) values) with respect to both 100% CF and 100% WF batters [3]. Moreover, batters with added XG were also more rigid (higher maximum complex modulus (G*_max_) values), this increase in rigidity being caused by an increase in elasticity (*G*′), as indicated by their lower loss tangent values (tan *δ*). This means that batters made with XG, especially at the highest concentration (1% XG batter), were denser than both control and 100% CF batters. The kinetic process occurring between the minimum temperature and the maximum temperature at each muffin batter was subsequently investigated with the non-isothermal kinetic model.

### 3.2. Kinetic Modeling of Elastic Modulus during Non-Isothermal Heating of CF-Based Gluten-Free Muffin Batters with Added Biopolymers

The flour gelatinization process can be quantified rheologically under SAOS tests and, further, it is reported that the gelatinization kinetics is best studied at relatively lower rate of heating [33]. Considering these points, the non-isothermal heating of muffin batters (control gluten and CF-based), both temperature and duration of heating time (1.6 °C·min^−1^) were considered. The reaction kinetics of dough/starch gelatinization was also studied based on non-isothermal heating since isothermal heating of dough/starch is affected by both temperature and duration of heating [22]. Earlier, the technique was applied to rice and lentil starch gelatinization [19,20], as well as to unpressurized and pressurized chickpea slurries [24] and batters [18]. In this study, the temperature range for kinetic analysis was selected from 25 °C up to 90 °C, which was, therefore, considered as the temperature where *G*′ achieved its maximum value. 

As described by Ahmed et al. [22] and Yoon et al. [34], the kinetic equation can be converted to Equation (6) in terms of rheological parameters (*G*′ and *dG*′) instead of reactant concentration (*C*) and change in concentration (*dC*), respectively, although, in the strict sense, the evolved elastic modulus is not linearly related to concentration [22]. The negative sign of kinetic Equation (4) is substituted by a positive sign because of the increase in *G*′ during heating (positive *dG*′):

(6) $$\frac{dG\prime }{dt}=k_{0}G\prime^{n}e^{\frac{-Ea}{RT}}$$

In addition, Equation (6) can be rewritten as Equation (7) [25]:

(7) $$\ln\left(\frac{1}{G\prime^{n}}\frac{dG\prime }{dt}\right)=\ln k_{0}-\left(\frac{E_{a}}{R}\right)\left(\frac{1}{T}\right)$$

Among various kinetic models, the non-isothermal technique is considered the best one since most of the gelatinization experimented in situ at a constant heating rate and variation in *G*′ measures the structure development rate (*dG*′/*dt*) [34]. Derivatives of experimental data are usually calculated by the following methods: (1) graphical differentiation; (2) polynomial curve fitting and differentiation of the fitted equation; and (3) numerical differentiation [26]. In this study, derivatives of experimental data were calculated from sixth-order polynomial fits (Equation (8)), approximating the change of *G*′ versus temperature (only for this fit *T* is expressed in °C) (for all of the cases, the corresponding mean *R*^2^ value was 0.998 ± 0.001) from 25°C to 90 °C.

(8) $$G\prime \approx C_{0}+C_{1}T+C_{2}T^{2}+C_{3}T^{3}+C_{4}T^{4}+C_{5}T^{5}+C_{6}T^{6}=\sum_{n=0}^{n=6}C_{n}T^{n}$$

**Figure 2:**
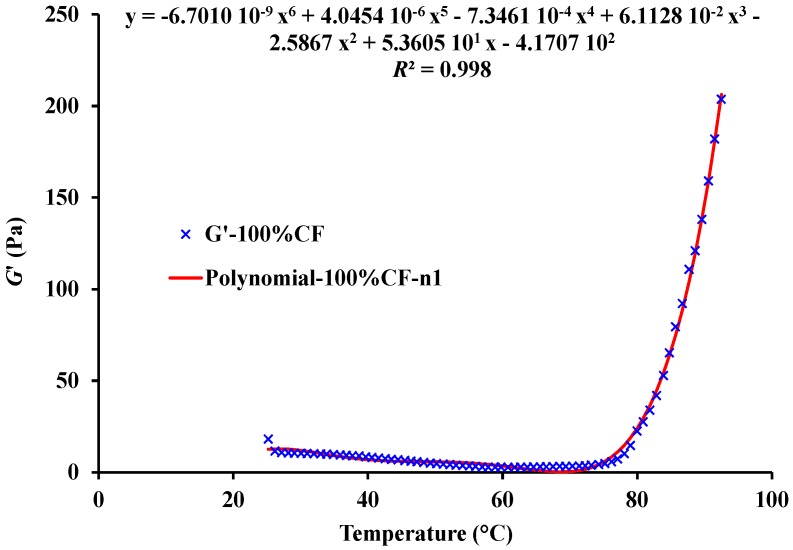
Sixth-order polynomial fit approximating the change of elastic modulus (*G*′) vs. temperature between 25 and 90 °C during non-isothermal heating of muffin batter made with chickpea flour (CF) alone (100% CF).

As an example, Figure 2 shows the sixth-order polynomial fit approximating the change of *G*′ vs. temperature during non-isothermal heating of a single sample of 100% CF muffin batter. In turn, Table 2 gives the mean coefficient values (*n* = 9) of sixth-order polynomial fits derived from the curves of *G*′ obtained for each batter. In a previous study, the derivatives of experimental data of various CF-based muffin batters were also calculated by sixth-order polynomial fits approximating the change of *G*′ versus time (*t*) [25].

As it can be seen in Figure 2, *G*′ decreases with temperature and exhibits a minimum value that is followed by a continuous increase upon further heating. In this study, the temperature (*T*_0_) which corresponds to the minimum value of the *G*′ before starting to increase was nominated as the inflexion temperature.

To approximate *dG*′/*dt* by deriving in Equation (8):

(9) $$\frac{dG\prime }{dt}=\frac{dG\prime }{dT}\frac{dT}{dt}\approx \frac{dT}{dt}\sum_{k=0}^{k=6}kC_{k}T^{\left(k-1\right)}$$

As the temperature increases linearly with time (*t*) during non-isothermal heating, one can write:

(10) T=A+Bt

where *A* = intercept term (°C) and *B* = heating rate (either °C·s^−1^ or K·s^−1^).

By deriving in Equation (10), (*dT*/*dt*) is expressed as:

(11) $$\frac{dT}{dt}=B$$

By substituting Equation (11) in Equation (9), the derivative *dG*′/*dt* can be approximated as follows:

(12) $$\frac{dG\prime }{dt}\approx B\sum_{k=0}^{k=6}kC_{k}T^{\left(k-1\right)}={\left(\frac{dG\prime }{dt}\right)}_{Experimental}$$

Equation (12) is considered as the experimental derivative.

**Figure 3:**
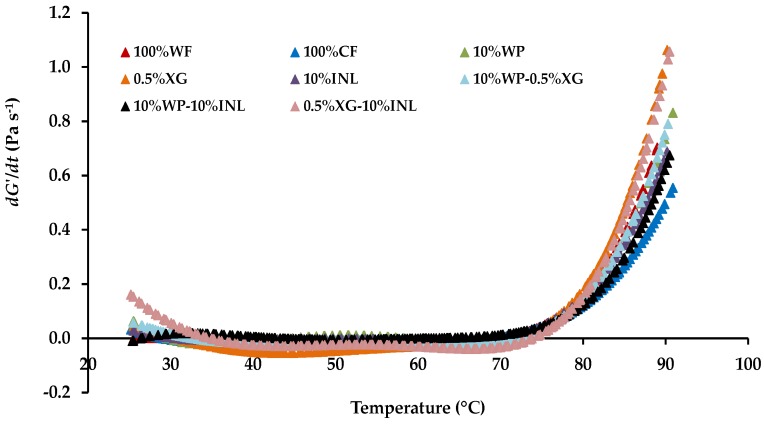
Variation of *dG*′/*dt* as a function of increasing temperature in the muffin control batter and batters with wheat flour (WF) replaced by chickpea flour (CF) and various percentages of biopolymers. WP, whey protein; XG, xanthan gum; INL, inulin. Mean values of nine measurements.

Figure 3 shows the variation of *dG*′/*d*t (Pa·s^−1^) versus temperature (°C) for the control WF-based batter and CF-based batters prepared with CF alone, with partial replacement of CF with WP, XG, and INL alone at intermediate levels and with their blends. For all the muffin batters, *dG*′/*d*t decreases with temperature and exhibits a minimum value that is followed by a continuous increase upon further heating and which is substantially coincident with the inflexion temperature *T*_0_. As stated above, in this study the temperature (*T*_0_) corresponding to the minimum value of the *G*′ before starting to increase was nominated as inflexion temperature. In this *T*_0_, starch granules start to swell (initial stage), leading to an increase of their volume and becoming closely packed in the system.

However, note that *T*_0_ is always lower than the *T*_gel_ used by other authors [24,25], and at which *G*′ and *G*′′ intersect. In a previous study, *T*_0_ was considered as the end point for the first downward curve and the beginning of the second curve, being the inflection point of the experimental curve *dG*′/*dt* vs. temperature [25]. The authors just cited proposed a multistep mechanism for the temperature-induced gelatinization of high hydrostatic pressure (HHP)-treated CF slurry. 

Both Figure 2 and Figure 3 show that the derivative *dG*′/*dt* is negative or null, especially at first, until a certain temperature is reached, from which continuously rises until the end of the process. For this reason, the kinetic model (Equation (6)) will be extended to the Equation (13):

(13) $$\frac{1}{G\prime^{n}}\frac{dG\prime }{dt}=a+k_{0}e^{-\frac{E_{a}}{RT}}$$

where *a* (Pa·s^−1^) is a constant avoiding the negative and null values of *dG*′/*dt*.

Nevertheless for sake of convenience in the non-linear regression, a kinetic equation with four constants (*a*, *b*, *c* and *d*) were considered as a better kinetic model for this paper. Therefore, Equation (13) remains:

(14) $$\frac{1}{G\prime^{n}}\frac{dG\prime }{dt}=a+be^{-c\left(\frac{1}{T}-d\right)}$$

At this point, it is to be mentioned that in all cases one trial and error method was performed to determine the order of the reaction. The procedure was as follows: the value of *n* was fixed. With this constant value of *n* the corresponding non-linear regression of Equation (14) was performed. In all cases the minimum standard error and the maximum correlation coefficient was obtained for *n* = 0. So, henceforth, we consider that this is a zero-order reaction and thus the factor *G*′*^n^* will be omitted.

Limited information is available on zero-order reaction kinetics for starch gelatinization. The gelatinization process of CF slurry in a narrow temperature range (from gel point to *G*′_max_), determined and averaged to be only about 14.3 ± 1.3 °C, followed zero-order kinetics well [24]. Analogously, zero-order reaction kinetics was also considered in five different muffin batters prepared with WF or CF alone and their blends [18], as well as in the case of high hydrostatic pressure treated CF slurries [25].

In contrast, the starch gelatinization process of mung bean starch individually and sample incorporating 10% sodium chloride and 10% sucrose [22], followed first-order kinetics well, with *n* = 1. However, addition of 5% sodium chloride, 5% sucrose, and their blend followed second-order gelatinization kinetics, i.e., *n* = 2. The temperature range selected by Ahmed [21] for kinetic analysis was from 50 °C to the *G*′_max_ value. Second-order reaction kinetics have also been reported for protein gelation in a wide temperature range from 20 °C to 80 °C [34]. 

Consequently, Equation (14) remains:

(15) $$\frac{dG\prime }{dt}=a+be^{-c\left(\frac{1}{T}-d\right)}$$

where *d* = 1/*T*_0_ (K^−1^), and *T*_0_ (K) is the absolute reference temperature as mentioned above. Parameter *a* (Pa·s^−1^) is the previously-mentioned constant avoiding the negative and null values of *dG*′/*dt*, whereas *b* (Pa·s^−1^) is other constant that, together with *T*_0_, represents the frequency factor *k*_0_ for the case of zero-order kinetics (*n* = 0) (as seen below in Equation (17)).

Thus, replacing *d* by 1*/T*_0_ in Equation (15), this can be converted into Equation (16) as follows:

(16) $$\frac{dG\prime }{dt}=a+be^{-c\left(\frac{1}{T}-\frac{1}{T_{0}}\right)}={\left(\frac{dG\prime }{dt}\right)}_{Theoretical}$$

Finally, comparing with Equation (13), one can obtain the corresponding frequency factor *K_0_* (Pa·s^−1^) (Equation (17)) and the activation energies (kJ·mol^−1^) (Equation (18)):

(17) $$k_{0}=be^{\frac{c}{T_{0}}}$$

(18) Ea=cR

In this point is to be mentioned that, as seen later in this paper and according to Lai et al. [35], there is a linear relationship between ln*k*_0_ and *E_a_*, so that it will actually be reduced to a bi-parametric second addend of Equation (16).

Nevertheless, it was also verified if the derivative of *G*′ with respect to *t* could be better approximated as the sum of various reaction processes (Equation (19)), each one in the form of Equation (16). In fact, Equation (16) can be rewritten as:

(19) $$\frac{dG\prime }{dt}=a_{1}+b_{1}e^{-c_{1}\left(\frac{1}{T}-\frac{1}{T_{0}}\right)}+a_{2}+b_{2}e^{-c_{2}\left(\frac{1}{T}-\frac{1}{T_{0}}\right)}+...$$

By fixing the *T*_0_ value as previously said and calculating the first difference between experimental and theoretical models as follows:

(20) $$Difference_{1}={\left(\frac{dG\prime }{dt}\right)}_{Experimental}-{\left(\frac{dG\prime }{dt}\right)}_{Theoretical1}=a_{2}+b_{2}e^{-c_{2}\left(\frac{1}{T}-\frac{1}{T_{0}}\right)}+...$$

Then, the calculation for the second regression is performed similarly, with fixed *d* (=1/*T*_0_) value, i.e., as:

(21) $$\frac{dG\prime }{dt}={\left(\frac{dG\prime }{dt}\right)}_{Theoretical2}={\left(\frac{dG\prime }{dt}\right)}_{Theoretical1}+a_{2}+b_{2}e^{-c_{2}\left(\frac{1}{T}-\frac{1}{T_{0}}\right)}+...$$

Again, the calculation for the second difference is performed similarly according to Equation (20):

(22) $$Difference_{2}={\left(\frac{dG\prime }{dt}\right)}_{Experimental}-{\left(\frac{dG\prime }{dt}\right)}_{Theoretical2}=a_{3}+b_{3}e^{-c_{3}\left(\frac{1}{T}-\frac{1}{T_{0}}\right)}+...$$

In this way, the process can be repeated successively.

**Figure 4:**
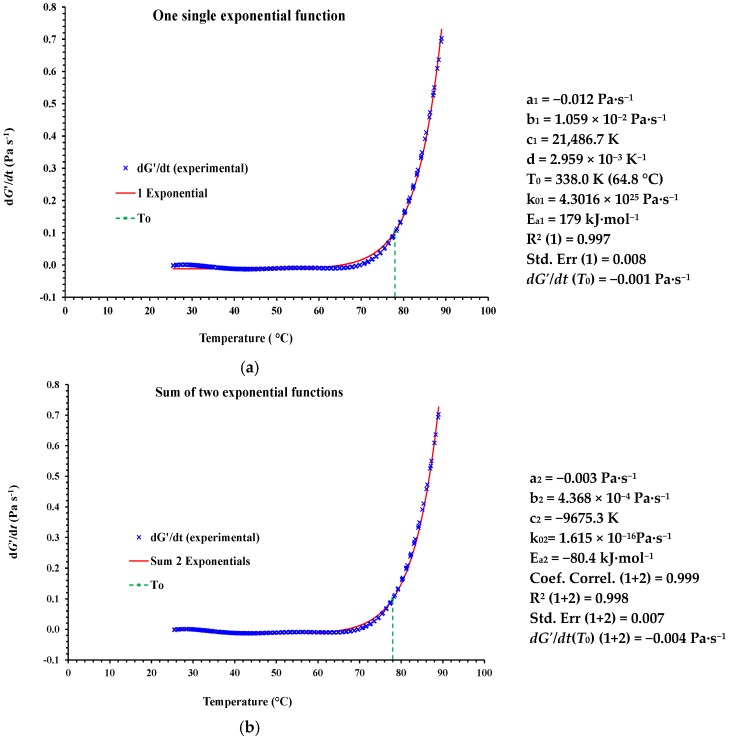
One single exponential function (**a**); and sum of two exponential functions (**b**) modeling the variation of the mean curve of *dG*′/*dt* versus temperature between 25°C and 90 °C in the muffin control batter (100% WF) together the kinetic parameters generated and the goodness of fits. Mean values of nine measurements (*n* = 9).

As an example, for purposes of comparison, the Figure 4 shows the fits approximating the change of the *dG*′/*dt* vs. temperature for the control WF batter using either a single exponential function or a the sum of two exponential functions. The kinetic parameters derived for both fits and *n* = 0 are also shown. In this example, the fits correspond to the mean curve of the change of the *dG*′/*dt* vs. temperature.

The need for using two exponential functions for describing the structure development rate (*dG*′/*dt*) can be interpreted as indicating that two reactions occurred, because of the existence of two phases. Rubens et al. [36] studied the gelatinization of starches under pressure in situ with Fourier-transform infrared spectroscopy. Analysis of the spectra led to the proposition of a multistep gelatinization mechanism, similar to the temperature-induced gelatinization. A two-step mechanism of HHP-induced gelatinization was further confirmed by Blaszczak et al. [37], who studied the gelatinization of potato starch treated at 600 MPa. This two-step mechanism was also in agreement with the study of Ahmed et al. [23], who observed similar two phases for the temperature induced gelatinization of wheat flour dough incorporating water insoluble date fiber. In that study, *G*′ increased abruptly with temperature, exhibiting a peak value at approximately 67 °C, which was followed by a continuous decrease up to 95 °C. Therefore, the authors divided the complete gelatinization curve (*G*′-temperature) into two parts: “up curve” (pre-gelatinization, swelling dominant) and “down curve” (gelatinization, disruption and leaching of the amylose, as well as amylopectin breakdown). A two-step mechanism for the heat-induced gelatinization process of HHP-treated CF slurry was also observed from the change of the derivative of elastic modulus with respect to *t* vs. temperature [25]. 

In the present study, for the control muffin batter (100% WF), by considering that only one reaction occurred (Figure 4a), the goodness of the fit for Equation (16) was very high, with the coefficient of determination (*R*^2^) and standard error (SE) of 0.997 and 0.008, respectively. The average value for the activation energy (*E*_a_) was found to be 179 kJ·mol^−1^. The other kinetic parameter obtained from the Equation (16) is *k_0_* = 4.30 × 10^25^ Pa·s^−1^. In turn, from the sum of the two reaction processes (Figure 4b), the corresponding *R*^2^ and SE values for the second exponential function in Equation (19) were 0.998 and 0.007, respectively. Therefore, the coefficient of determination (*R*^2^) and the SE of the second reaction process were slightly higher and lower, respectively, compared with those corresponding to the first reaction process. Similar results were obtained for all the CF-based batters with and without added biopolymers and their blends. By considering the fits for the mean curves at each case, the *R*^2^ and SE values for single exponential functions (Equation (16)) ranged between 0.985 and 0.999, and between 0.007 and 0.063, respectively. As could be expected from the shape of the curves (Figure 1b), the worst fits of experimental data for Equation (16) corresponded to the muffin batters with XG added at 1% probably due to the increase in rigidity observed in this sample. In turn, the *R*^2^ and SE values for the second exponential functions from Equation (19) ranged between 0.995 and 0.999, and between 0.011 and 0.033, respectively. Since the calculated value of *R*^2^ was nearly equal to one in both cases, and for simplicity, a single exponential function was considered for describing the muffin batters gelatinization process.

Therefore, in this study, it was considered that the Equation (16) was sufficient to fully reflect the actual evolution of the gelatinization and coagulation processes taking place in either the 100% WF control or the CF-based gluten-free muffin batters with and without added biopolymers under non-isothermal heating. The values obtained for the constants (*a*, *b*, and *c*), the inflection temperature (*T*_0_), as well as the kinetic parameters (*k*_0_ and *E*_a_) are shown in Table 3. The *d* values have been omitted for the sake of brevity, as it is the inverse of the absolute inflection temperature (*T*_0_).

The *a* values from Equation (16) ranged between −0.007 and −0.054 Pa·s^−1^ for 10% WP-10% INL and 1% XG batters, respectively. Nevertheless, as mentioned above, *a* only absorbs the negative values of (*dG*′/*dt*)_Experimental_, and it is not considered a kinetic parameter by itself. In turn, the *b* values ranged between 0.007 and 0.102 Pa·s^−1^. The highest significant *b* value corresponded to CF-based batter prepared with partial replacement of CF with XG at 0.5% level blended with INL at 10% level.

One-hundred percent WF and 15% INL muffin batters showed the highest *c* values without significant differences between them. With regard to the *T*_0_ values, there were no significant differences in the inflection temperature determined in the muffins prepared with partial replacement of CF with XG alone at 1% level (1% XG batter) and with and with partial replacement of CF with XG at 0.5% level blended with INL at 10% level (0.5% XG-10% INL), and they had the highest *T*_0_ values, which could be explained as these samples showed the highest batter density (Figure 1b,d) and, therefore, enabling them more difficult to gelatinize. The increased batter viscoelasticity in the presence of jambolan fruit pulp (JFP) and XG was also attributed to their high water-binding capacity, which might have made free water unavailable [15]. This property has been attributed to the hydroxyl groups in the XG structure, which allow more hydrogen bonding with water molecules [38]. A previous study also showed that the incorporation of XG produced a delay in the *T*_gel_, which was ~79 ± 2.3 °C for 1% XG batter and 78.5 ± 5.0 °C for 0.5% XG-10% INL [3]. However, it should be note that, as mentioned previously, the *T*_0_ meaning in Equation (16) is different from the cross-over temperature (*T*_gel_) where the *G*′ and *G*′′ moduli intersect. The delay produced by incorporation of XG would allow starch gelatinization to occur later, giving enough time for appropriate air and vapor expansion during heating and avoiding thermosetting [13,14]. In contrast, the batters with WP alone (at 5% and 10%) showed the lowest *T*_0_ values, which could be explained as the presence of WP alone produced weaker batters. On the other hand, there were no significant differences in the *T*_0_ determined in both 100% WF and 100% CF batters.

The magnitude of frequency factors ranged between 6.25 × 10^16^ and 5.78 × 10^25^ Pa·s^−1^. For this kinetic parameter, the geometric means are shown in the Table 3. The highest *k*_0_ value corresponded to control gluten 100% WF batter, whereas the lowest one was found for the batter with partial replacement of CF with INL at the lowest level (5% INL). Similar values of frequency factor (1.54 × 10^19^–1.70 × 10^26^) were reported for starch gelatinization reaction kinetics during non-isothermal heating of water insoluble date fiber incorporated wheat flour dough [23].

The activation energy (*E*_a_) of the reaction has been considered as the parameter essential from the point of view of the investigated mechanism of the reaction [39]. In this study, the *E*_a_ values of muffin batters varied with formulation ranging between 118 and 180 kJ·mol^−1^. The highest *E*_a_ corresponded to control gluten 100% WF batter (Table 3). However, no significant differences were found in *E*_a_ values between the control (180 kJ·mol^−1^) and 15% INL (163 kJ·mol^−1^). The higher *E*_a_ at 100% WF and 15% INL batters would imply that they were less favorable for gelatinization [24,34]. 100% CF batter showed significantly (*p* < 0.05) lower *E*_a_ than the control batter, which also indicates that batter made with CF alone was more favorable for gelatinization, so that it needs a lower energy requirement to achieve critical gel rigidity [22]. The significant increase in *E*_a_ values for gelatinization of control sample could be attributed by competition of water availability among ingredients. It may be because of the higher energy required to gelatinize higher starch content in case of 100% WF batter, as well as gluten presence. According to the supplier, the total carbohydrate content of CF is 55% *w*/*w*, whereas lower starch content ranging between 34.9% and 42.9% *w*/*w* has been reported for CF [40]. On the other hand, higher both carbohydrate (85% *w*/*w*) and starch (78.8% *w*/*w*) contents have been reported for WF [41]. Most likely, the different proportions of wheat and chickpea proteins present in the cellular system also modified the viscoelasticity of the CF-based muffin batter during non-isothermal heating, as well as the kinetic parameters derived. In this study, protein contents provided by the supplier were 10.2% and 19.4% for WF and CF, respectively. In turn, gluten proteins (ca. 80%–85% of total wheat protein) are the main storage proteins of wheat [42] with valuable hydration properties. Upon hydration and mixing, they form a strong, cohesive, viscoelastic network that allows the WF dough to retain yeast fermentation gases and to produce a light, aerated baked product.

Regarding the addition of the various biopolymers, replacement of CF with WP or XG alone at any concentration, INL at 5% and 10% and the three blends of ingredients hardly modified the *E*_a_ of the 100% CF batter. Therefore, only INL at the highest concentration (15% INL) produced a significant increase in this kinetic parameter. In the same way, 15% INL produced a slight improvement in the viscoelastic properties, as reflected by their significantly (*p* < 0.01) lower tan δ value and higher moduli with respect to 100% WF and 100% CF batters [3].

In the literature, a wide range of *E*_a_ values (10–964 kJ·mol^−1^) has been reported. Yoon and Gunase karan [43] reported *E*_a_ of 118 to 179 kJ·mol^−1^ for the gelation of xanthan and carob mixtures. The magnitudes of *E*_a_ were 32.2 and 26.1 kJ·mol^−1^ during non-isothermal heating of rice and mung bean starch [21,22]. A lower *E*_a_ for CF slurries (~20 kJ·mol^−1^) is because of considering a narrower temperature range for the starch gelatinization process [24]. Probably, the different methods used by the different authors for calculating derivatives of experimental data influenced the *E*_a_ values reported in the literature. A simultaneous and sequential estimation of kinetic parameters in a starch viscosity model was also carried out by Sulaiman et al. [44]. The authors just cited found a higher *E*_a_ value (964 kJ·mol^−1^) compared to other values reported. Similarly, Dolan and Steffe [45] developed a complete starch model for rheological behavior of gelatinizing starch solutions with a high *E*_a_ value of 740 kJ·mol^−1^. The large difference of *E*_a_ might be due to different botanical starches having different starch chemistry and different methods of gelatinization that were used [44].

The relationships between coefficients and kinetic parameters derived from Equation (16) were established as shown in Table 4. The highest significant negative correlation between coefficients was found between *a* and *b* constants. In this study, the higher *E*_a_ found in the control gluten batter was also accompanied by a parallel higher *k*_0_ value. In fact, there was a significant positive correlation (*r* = 0.760) between the *k*_0_ and *E*_a_ values derived for each muffin batter.

It is known that, for a structurally-related series of compounds undergoing a defined chemical reaction, parallel changes in enthalpy and entropy are usually found [35]. This is the so-called enthalpy-entropy compensation effect and can alternatively be described by the systematic variation of frequency factor with activation energy for the same family of reactions, where the *k*_0_ and *E*_a_ are usually derived by Arrhenius’ laws. This relationship is also named an isokinetic relationship (IKR), because the resultant slope suggests an isokinetic temperature at which the reaction rate constant is identical for all processes concerned [26,46,47]. The existence of an IKR implies that only one reaction mechanism is followed by all members of the reaction series [35]. There was a linear IKR between ln *k*_0_ and *E*_a_ data given by Lai et al. [35], with *R*^2^ = 0.906. And as mentioned above, in the present study, there was also a significant linear relationship between the ln*k*_0_ and *E*_a_ values (Equation (23)) with *R*^2^ = 0.998:

(23) $$\ln k_{0}=-1.2105+0.3377\;E_{a}$$

Ignoring *a*, this reduces the three parametric second addend of Equation (16) into a bi-parametric one. In fact, Equation (23) can also be written as:

(24) $$k_{0}=e^{-1.2105}\times e^{0.3377E_{a}}=0.2981\times e^{0.3377E_{a}}$$

denominating $T_{0}$:

(25) $$d=1/T_{0}=\frac{10^{3}}{0.3377R}\approx \frac{1}{356.2}$$

Substituting Equations (24) and (25) in Equation (15), and rearranging terms, this becomes:

(26) $$\frac{dG\prime }{dt}=a+0.2981\times e^{-\frac{E_{a}}{R}\left(\frac{1}{T}-\frac{1}{T_{0}}\right)}$$

where *E*_a_ is expressed in kJ·mol^−1^ and parameters *b* = 0.2981 and *T*_0_ = 356.2 K are two process constants and not two parameters dependent on the particular batter.

## 4. Conclusions

Therefore, an apparent kinetic compensation effect was also observed for the temperature-induced gelatinization of muffin batters, which could be explained by associated granular structural changes occurring during heating. However, more systematic studies may be needed to find out the relationship between *G*′ and kinetic parameters.

Nevertheless, remarkably, the activation energies seemed to be good indicators of the degree of gelatinization induced in the muffin batters by the non-isothermal heating when a 1.6 °C·min^−1^ heating ramp is employed from 25°C to 90 °C. CF-based gluten-free batters with starch and protein contents closer to the levels of gluten WF-based batter could be a strategy to decrease differences in kinetic parameters of muffin batters, looking for *E*_a_ values close to that of the control gluten batter, i.e., ~180 kJ·mol^−1^.

## Figures and Tables

**Table 1:** Formulations of CF-based gluten-free muffin batters (g 100 g^−1^ flour or flour-biopolymer or flour-biopolymer blends).

Formulation	100% WF	100% CF	5% WP	10% WP	15% WP	0.25% XG	0.5% XG	1% XG	5% INL	10% INL	15% INL	10% WP-0.5% XG	10% WP-10% INL	0.5% XG-10% INL
WF	100	0	0	0	0	0	0	0	0	0	0	0	0	0
CF	0	100	95	90	85	99.75	99.5	99	95	90	85	89.5	80	89.5
WP	0	0	5	10	15	0	0	0	0	0	0	10	10	0
XG	0	0	0	0	0	0.25	0.50	1	0	0	0	0.50	0	0.50
INL	0	0	0	0	0	0	0	0	5	10	15	0	10	10
Whole egg	81	81	81	81	81	81	81	81	81	81	81	81	81	81
Sucrose	100	100	100	100	100	100	100	100	100	100	100	100	100	100
Salt	0.75	0.75	0.75	0.75	0.75	0.75	0.75	0.75	0.75	0.75	0.75	0.75	0.75	0.75
Milk	50	50	50	50	50	50	50	50	50	50	50	50	50	50
Oil	46	46	46	46	46	46	46	46	46	46	46	46	46	46
Citric acid	3	3	3	3	3	3	3	3	3	3	3	3	3	3
Sodium hydrogen carbonate	4	4	4	4	4	4	4	4	4	4	4	4	4	4

WF, wheat flour; CF, chickpea flour; WP, whey protein; XG, xanthan gum; INL, inulin.

**Table 2:** Mean coefficients of sixth-order polynomial fits obtained approximating the change of elastic modulus (*G*′) vs. temperature for each CF-based gluten-free muffin batter together 100% WF control.

Coefficients/Codes	C_6_	C_5_	C_4_	C_3_	C_2_	C_1_	C_0_
**100% WF**	5.222 × 10^−8^	−1.491 × 10^−5^	1.728 × 10^−3^	−1.035 × 10^−1^	3.357 × 10^0^	−5.586 × 10^1^	3.950 × 10^2^
**100% CF**	2.467 × 10^−9^	1.139 × 10^−6^	−3.849 × 10^−4^	4.127 × 10^−2^	−2.063 × 10^0^	4.876 × 10^1^	−4.223 × 10^2^
**5% WP**	−6.056 × 10^−9^	5.058 × 10^−6^	−1.043 × 10^−3^	9.468 × 10^−2^	−4.311 × 10^0^	9.585 × 10^1^	−8.146 × 10^2^
**10% WP**	−3.198 × 10^−9^	4.844 × 10^−6^	−1.113 × 10^−3^	1.064 × 10^−1^	−4.999 × 10^0^	1.134 × 10^2^	−9.792 × 10^2^
**15% WP**	−3.842 × 10^−9^	4.168 × 10^−6^	−9.097 × 10^−4^	8,497 × 10^−2^	−3.940 × 10^0^	8.883 × 10^1^	−7.628 × 10^2^
**0.25% XG**	1.366 × 10^-8^	−8.840 × 10^−7^	−3.249 × 10^−4^	5,107 × 10^−2^	−2.949 × 10^0^	7.596 × 10^1^	−6.887 × 10^2^
**0.5% XG**	7.487 × 10^−8^	−2.126 × 10^−5^	2.404 × 10^−3^	−1.359 × 10^−1^	3.921 × 10^0^	−5.243 × 10^1^	3.201 × 10^2^
**1% XG**	2.891 × 10^−8^	−5.557 × 10^−6^	2.002 × 10^−4^	2.757 × 10^−2^	−2.875 × 10^0^	9.681 × 10^1^	−9.078 × 10^2^
**5% INL**	2.371 × 10^−9^	1.709 × 10^−6^	−5.122 × 10^−4^	5.208 × 10^−2^	−2.489 × 10^0^	5.664 × 10^1^	−4.839 × 10^2^
**10% INL**	1.294 × 10^−8^	−1.884 × 10^−6^	−2.126 × 10^−5^	1.774 × 10^−2^	−1.202 × 10^0^	3.233 × 10^1^	−3.020 × 10^2^
**15% INL**	1.382 × 10^−7^	−4.424 × 10^−5^	5.757 × 10−^3^	−3.880 × 10^−1^	1.421 × 10^1^	−2.674 × 10^2^	2.036 × 10^3^
**10% WP-0.5% XG**	2.880 × 10^−8^	−6.761 × 10^−6^	5.736 × 10^−4^	−1.829 × 10^−2^	−1.099 × 10^−1^	1.784 × 10^1^	−1.796 × 10^2^
**10% WP-10% INL**	6.017 × 10^−8^	−1.866 × 10^−5^	2.371 × 10^−3^	−1.569 × 10^−1^	5.669 × 10^0^	−1.050 × 10^2^	7.910 × 10^2^
**0.5% XG-10% INL**	4.190 × 10^−8^	−9.414 × 10^−6^	6.871 × 10^−4^	−7.748 × 10^−3^	−1.292 × 10^0^	5.842 × 10^1^	−6.162 × 10^2^

WF, wheat flour; CF, chickpea flour; WP, whey protein; XG, xanthan gum; INL, inulin; C_6_, sextic coefficient; C_5_, quintic coefficient; C_4_, quartic coefficient; C_3_, cubic coefficient; C_2_, quadratic coefficient; C_1_, linear coefficient; C_0_, constant.

**Table 3:** Coefficients and kinetic parameters obtained approximating the change of the derivative of elastic modulus (*G*′) with respect to *t* by using Equation (16) for each CF-based gluten-free muffin batter together 100% WF control.

Codes	*a* (Pa·s^−1^)	*b* (Pa·s^−1^)	*c* (K)	*T*_0_ (°C)	*k*_0_ (Pa·s^−1^)	*E*_a_ (kJ·mol^−1^)	*R*^2^	*SE*
**100% WF**	−0.012 ± 0.003^A,B^	0.012 ± 0.008^B^	21,591 ± 2670^A^	64.8 ± 1.96^B−D^	5.78 × 10^25^	180 ± 22.2^A^	0.994 ± 0.002	0.011 ± 0.001
**100% CF**	−0.013 ± 0.001^A−C^	0.018 ± 0.006^B^	17,075 ± 1512^C^	65.6 ± 3.66^B−D^	1.33 × 10^20^	142 ± 12.6^C^	0.993 ± 0.003	0.013 ± 0.001
**5% WP**	−0.016 ± 0.005^A−C^	0.010 ± 0.004^B^	15,701 ± 1539^C,D^	57.9 ± 2.19^G^	3.77 × 10^18^	131 ± 12.8^C,D^	0.994 ± 0.003	0.027 ± 0.012^B−E^
**10% WP**	−0.022 ± 0.002^B−D^	0.016 ± 0.002^B^	15,834 ± 737^C,D^	60.8 ± 1.10^E−G^	6.17 × 10^18^	132 ± 6.13^C,D^	0.995 ± 0.002	0.036 ± 0.008
**15% WP**	−0.016 ± 0.002^A−C^	0.018 ± 0.006^B^	15,207 ± 1440^C,D^	62.1 ± 1.80^D−F^	8.85 × 10^17^	126 ± 12.0^C,D^	0.994 ± 0.002	0.029 ± 0.005
**0.25% XG**	−0.023 ± 0.008^C−E^	0.029 ± 0.019^B^	16,249 ± 2666^C,D^	63.6 ± 2.50^B−E^	2.24 × 10^19^	135 ± 22.2^C,D^	0.994 ± 0.003	0.033 ± 0.014
**0.5% XG**	−0.043 ± 0.004^F^	0.040 ± 0.007^B^	17,743 ± 1353^B,C^	67.3 ± 1.35^B^	1.70 × 10^21^	148 ± 11.2^B,C^	0.996 ± 0.002	0.033 ± 0.006
**1% XG**	−0.054 ± 0.014^G^	0.078 ± 0.033^A^	16,396 ± 1753^C,D^	71.1 ± 1.31^A^	3.60 × 10^19^	136 ± 14.6^C,D^	0.983 ± 0.011	0.065 ± 0.029
**5% INL**	−0.019 ± 0.003^B−D^	0.024 ± 0.001^B^	14,224 ± 351^D^	62.3 ± 1.00^C−E^	6.25 × 10^16^	118 ± 2.92^D^	0.992 ± 0.002	0.029 ± 0.005
**10% INL**	−0.019 ± 0.001^B−D^	0.025 ± 0.003^B^	15,413 ± 600^C,D^	64.2 ± 0.058^B−E^	1.73 × 10^18^	128 ± 4.99^C,D^	0.996 ± 0.001	0.025 ± 0.002
**15% INL**	−0.016 ± 0.001^A−C^	0.014 ± 0.008^B^	19,663 ± 1385^A,B^	63.6 ± 1.53^C−E^	2.85 × 10^23^	163 ± 11.5^A,B^	0.998 ± 0.000	0.033 ± 0.003
**10% WP−0.5% XG**	−0.028 ± 0.010^D,E^	0.030 ± 0.004^B^	16,440 ± 1518^C,D^	65.9 ± 1.15^B,C^	3.39 × 10^19^	137 ± 12.6^C,D^	0.994 ± 0.002	0.034 ± 0.003
**10% WP−10% INL**	−0.007 ± 0.003^A^	0.007 ± 0.001^B^	17,667 ± 140^B,C^	59.7 ± 0.351^F,G^	8.16 × 10^20^	147 ± 1.16^B,C^	0.998 ± 0.000	0.018 ± 0.005
**0.5% XG−10% INL**	−0.033 ± 0.012^E,F^	0.102 ± 0.063^A^	17,471 ± 1500^B,C^	72.8 ± 5.27^A^	7.42 × 10^20^	145 ± 12.5^B,C^	0.989 ± 0.007	0.054 ± 0.009

Mean values (*n* = 9) ± standard deviation (SD). WF, wheat flour; CF, chickpea flour; WP, whey protein; XG, xanthan gum; INL, inulin; *a*, *b* and *c*, constants obtained from Equation (16); *T*_0_, inflection temperature; *k*_0_, pre-exponential or frequency factor; *E*_a_, activation energy; *R*^2^, determination coefficient; SE, standard error; ^A−G^ Mean values without the same letter are significantly different (*p* < 0.05).

**Table 4:** Matrix of correlations between coefficients and kinetic parameters.

	*a* (Pa·s^−1^)	*b* (Pa·s^−1^)	*c* (K)	*T*_0_ (°C)	*k*_0_ (Pa·s^−1^)	*E*_a_ (kJ·mol^−1^)
*a* (Pa·s^−1^)	1	−0.773 *	0.162	−0.740 *	0.255	0.162
*b* (Pa·s^−1^)	-	1	−0.159	0.890 *	−0.205	−0.159
*c* (K)	-	-	1	0.113	0.760 *	1.00 *
*T*_0_ (°C)	-	-	-	1	0.030	0.113
*k*_0_ (Pa·s^−1^)	-	-	-	-	1	0.760 *
*E*_a_ (kJ·mol^−1^)	-	-	-	-	-	1

*a*, *b* and *c*, constants obtained from Equation (16); *T*_0_, inflection temperature; *k*_0_, pre-exponential or frequency factor; *E*_a_, activation energy. * Significant correlations at *p* < 0.05 level.

## Supplementary Materials

Supplementary File 1

## References

[1] Matos M.E., Sanz T., Rosell C.M. (2014). Establishing the function of proteins on the rheological and quality properties of rice based gluten free muffins. Food Hydrocoll..

[2] Sanz T., Salvador A., Baixauli R., Fiszman S.M. (2009). Evaluation of four types of resistant starch in muffins. II. Effects in texture, colour and consumer response. Eur. Food Res. Technol..

[3] Herranz B., Canet W., Jiménez M.J., Fuentes R., Alvarez M.D. (2016). Characterisation of chickpea flour-based gluten-free batters and muffins with added biopolymers: Rheological, physical and sensory properties. Int. J. Food Sci. Technol..

[4] Gularte M.A., de la Hera E., Gómez M., Rosell C.M. (2012). Effect of different fibers on batter and gluten-free layer cake properties. LWT-Food Sci. Technol..

[5] Gularte M.A., Gómez M., Rosell C.M. (2012). Impact of legume flours on quality and in vitro digestibility of starch and protein from gluten-free cakes. Food Bioprocess Technol..

[6] Shevkani K., Kaur A., Kumar S., Singh N. (2015). Cowpea protein isolates: Functional properties and application in gluten-free rice muffins. LWT-Food Sci. Technol..

[7] Ronda F., Oliete B., Gómez M., Caballero P.A., Pando V. (2011). Rheological study of layer cake batters made with soybean protein isolate and different starch sources. J. Food Eng..

[8] Matos M.E., Rosell C.M. (2011). Chemical composition and starch digestibility of different gluten-free breads. Plant Food Hum. Nutr..

[9] Mariotti M., Lucisano M., Pagani M.A., Ng P.K.W. (2009). The role of corn starch, amaranth flour, pea isolate, and Psyllium flour on the rheological properties and the ultrastructure of gluten-free doughs. Food Res. Int..

[10] Walstra P., Dekker M. (2003). Soft solids. Physical Chemistry of Foods.

[11] Aguilar N., Albanell E., Miñarro B., Capellas M. (2015). Chickpea and tiger nut flours as alternatives to emulsifier and shortening in gluten-free bread. LWT-Food Sci. Technol..

[12] Miñarro B., Albanell E., Aguilar N., Guamis B., Capellas M. (2012). Effect of legume flours on baking characteristics of gluten-free bread. J. Cereal Sci..

[13] Martínez-Cervera S., Sanz T., Salvador A., Fiszman S.M. (2012). Rheological, textural and sensorial properties of low-sucrose muffins reformulated with sucralose/polydextrose. LWT-Food Sci. Technol..

[14] Martínez-Cervera S., de la Hera E., Sanz T., Gómez M., Salvador A. (2012). Effect of using erythritol as a sucrose replacer in making Spanish muffins incorporating xanthan gum. Food Bioprocess Technol..

[15] Singh J.P., Kaur A., Shevkani K., Singh N. (2015). Influence of jambolan (*Syzygium cumini*) and xanthan gum incorporation of the physicochemical and sensory properties of gluten-free eggless rice muffins. Int. J. Food Sci. Technol..

[16] Ureta M.M., Olivera D.F., Salvadori V.O. (2014). Baking of muffins: Kinetics of crust color development and optimal baking time. Food Bioprocess Technol..

[17] Martínez-Cervera S., Salvador A., Sanz T. (2014). Comparison of different polyols as total sucrose replacers in muffins: Thermal, rheological, texture and acceptability properties. Food Hydrocoll..

[18] Alvarez M.D., Herranz B., Fuentes R., Cuesta F.J., Canet W. (2016). Replacement of wheat flour by chickpea flour in muffin batter: Effect on rheological properties. J. Food Process Eng..

[19] Ahmed J., Ramaswamy H.S., Ayad A., Alli I., Alvarez P. (2007). Effect of high-pressure treatment on rheological, thermal and structural changes in Basmati rice flour slurry. J. Cereal Sci..

[20] Ahmed J., Auras R. (2011). Effect of acid hydrolysis on rheological and thermal characteristics of lentil starch slurry. LWT-Food Sci. Technol..

[21] Ahmed J. (2012). Rheometric non-isothermal gelatinization kinetics of mung bean starch slurry: Effect of salt and sugar—Part 1. J. Food Eng..

[22] Ahmed J., Ramaswamy H.S., Ayad A., Alli I. (2008). Thermal and dynamic rheology of insoluble starch from basmati rice. Food Hydrocoll..

[23] Ahmed J., Almusallam A.S., Al-Salman F., AbdulRahman M.H., Al-Salem E. (2013). Rheological properties of water insoluble date fiber incorporated wheat flour dough. LWT-Food Sci. Technol..

[24] Alvarez M.D., Fuentes R., Olivares M.D., Cuesta F.J., Canet W. (2014). Thermorheological characteristics of chickpea flour slurry as affected by moisture content. J. Food Eng..

[25] Alvarez M.D., Cuesta F.J., Fuentes R., Canet W. (2016). Rheometric non-isothermal gelatinization kinetics of high hydrostatic pressure treated chickpea flour slurry. J. Food Eng..

[26] Rhim J.W., Nunes R.V., Jones V.A., Swartzel K.R. (1989). Determinant of kinetic parameters using linearly increasing temperature. J. Food Sci..

[27] Shelke K., Faubion J.M., Hoseney R. (1990). The dynamics of cake baking as studied by a combination of viscosimetry and electrical resistance oven heating. Cer. Chem..

[28] Migliori M., Gabriele D., Baldino N., Lupi F.R., De Cindio B. (2011). Rheological properties of batter dough: Effect of egg level. J. Food Proc. Eng..

[29] Chen H.H., Kang H.Y., Chen S.D. (2008). The effects of ingredients and water content on the rheological properties of batters and physical properties of crusts in fried foods. J. Food. Eng..

[30] Gómez M., Oliete B., Rosell C.M., Pando V., Fernández E. (2008). Studies on cake quality made of wheat-chickpea flour blends. LWT-Food Sci. Technol..

[31] Baeza R.I., Carp D.J., Pérez O.E., Pilosof A.M.R. (2002). k-carrageenan-protein interactions: Effect of proteins on polysaccharide gelling and textural properties. Lebensm. Wiss. Technol..

[32] Meares C.A., Bogracheva T.Y., Hill S.E., Hedley C.L. (2004). Development and testing of methods to screen chickpea flour for starch characteristics. StarchStärke.

[33] Labropoulos A.E., Hsu S. (1996). Viscoelastic behavior of whey protein isolates at the sol-gel transition point. J. Food Sci..

[34] Yoon W.B., Gunasekaran S., Park J.W. (2004). Characterization of thermorheological behavior of Alaska pollock and Pacific whiting surimi. J. Food Sci..

[35] Lai V.M.-F., Lii C.-Y., Hung W.-F., Lu T.-J. (2000). Kinetic compensation effect in depolymerisation of food. Polysaccharides. Food Chem..

[36] Rubens P., Snauwaert J., Heremans K., Stute R. (1999). In situ observation of pressure-induced gelation of starches studied with FITR in the diamond anvil cell. Carbohydr. Polym..

[37] Blaszczak W., Valverde S., Fornal J. (2005). Effect of high pressure on the structure of potato starch. Carbohydr. Polym..

[38] Guarda A., Rosell C.M., Benedito C., Galotto M.J. (2004). Different hydrocolloids as bread improvers and antistaling agents. Food Hydrocoll..

[39] Malecki A., Prochowska-Klisch B., Wojciechowski K.T. (1998). Determination of the kinetics parameters of chemical reactions of the basis of non-isothermal measurement. J. Therm. Anal. Calorim..

[40] Xu Y., Thomas M., Bhardwaj H.L. (2014). Chemical composition, functional properties and microstructural characteristics of three kabuli chickpea (*Cicer arietinum* L.) as affected by different cooking methods. Int. J. Food Sci. Technol..

[41] Abebe W., Collar C., Ronda F. (2015). Impact of variety type and particle size distribution on starch enzymatic hydrolysis and functional properties of tef flours. Carbohydr. Polym..

[42] Van Der Borght A., Goesaert H., Veraverbeke W.S., Delcour J.A. (2005). Fractionation of wheat and wheat flour into starch and gluten: Overview of the main processes and the factors involved. J. Cer. Sci..

[43] Yoon W.B., Gunasekaran S., Fischer P., Marti I., Windhab E.J. (2000). Evaluation of structure development during gelation of xanthan and carob mixture. Proceedings of the 2nd International Symposium on Food Rheology and Structure.

[44] Sulaiman R., Dolan K.D., Mishra D.K. (2013). Simultaneous and sequential estimation of kinetic parameters in a starch viscosity model. J. Food Eng..

[45] Dolan K.D., Steffe J.F. (1990). Modeling rheological behaviour of gelatinizing starch solutions using mixer viscometry data. J. Texture Stud..

[46] Rhim J.W., Nunes R.V., Jones V.A., Swartzel K.R. (1989). Appearance of a kinetic compensation effect in the acid-catalyzed hydrolysis of disaccharides. J. Food Sci..

[47] Qin Z., Balasubramanian S.K., Wolkers W.F., Pearce J.A., Bischof J.C. (2014). Correlated parameter fit of Arrhenius model for thermal denaturation of proteins and cells. Ann. Biomed. Eng..

