# Supplementary figures and images for: Rheometric Non-Isothermal Gelatinization Kinetics of Chickpea Flour-Based Gluten-Free Muffin Batters with Added Biopolymers
Source: Foods. 2017 Jan 2;6(1):3. doi: 10.3390/foods6010003 (PMC5296672; doi:10.3390/foods6010003)

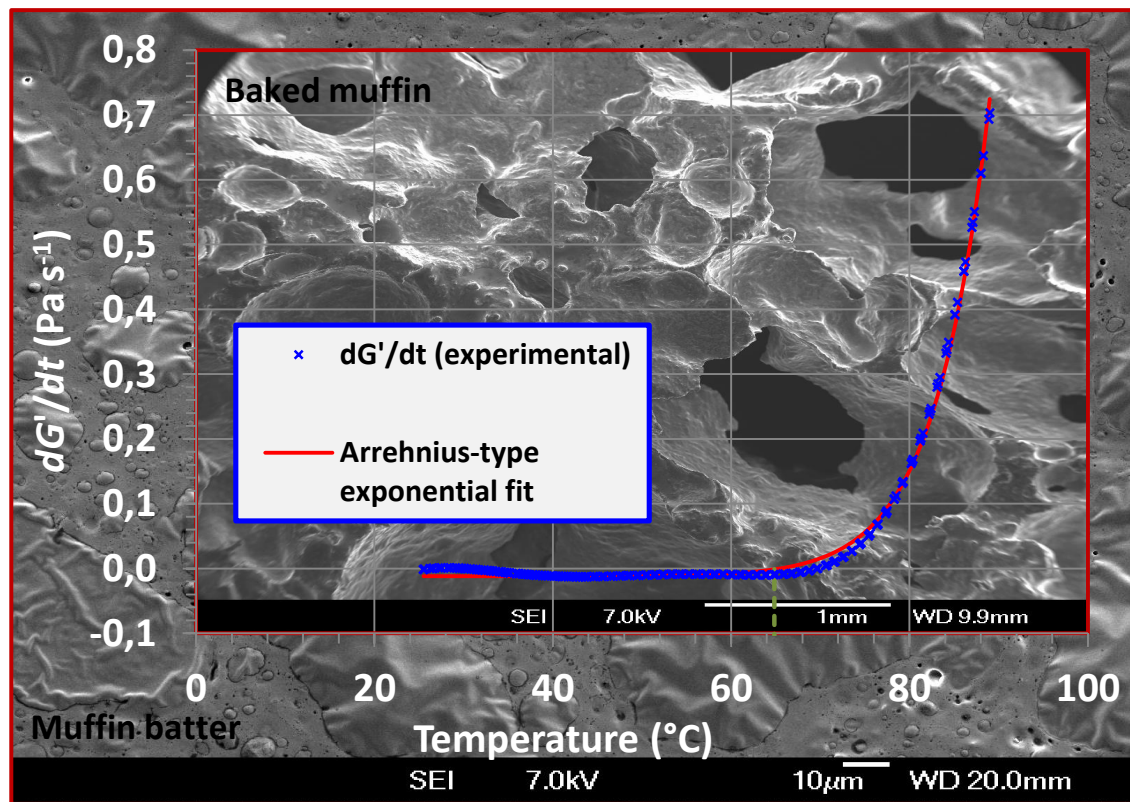

Supplement: Supplementary File 1 [file foods-06-00003-s001.pdf]
